# Progress in methods for evaluating Schwann cell myelination and axonal growth in peripheral nerve regeneration via scaffolds

**DOI:** 10.3389/fbioe.2023.1308761

**Published:** 2023-12-07

**Authors:** Jue Ling, Chang He, Shuxuan Zhang, Yahong Zhao, Meifeng Zhu, Xiaoxuan Tang, Qiaoyuan Li, Liming Xu, Yumin Yang

**Affiliations:** ^1^ Key Laboratory of Neuroregeneration, Ministry of Education and Jiangsu Province, Co-Innovation Center of Tissue Engineering and Nerve Injury Repair, Nantong University, Nantong, China; ^2^ College of Life Sciences, Nankai University, Tianjin, China; ^3^ Institute of Medical Device Control, National Institutes for Food and Drug Control, Beijing, China

**Keywords:** peripheral nerve injury, nerve regeneration, natural polymer, Schwann cell, neurotrophic factors

## Abstract

Peripheral nerve injury (PNI) is a neurological disorder caused by trauma that is frequently induced by accidents, war, and surgical complications, which is of global significance. The severity of the injury determines the potential for lifelong disability in patients. Artificial nerve scaffolds have been investigated as a powerful tool for promoting optimal regeneration of nerve defects. Over the past few decades, bionic scaffolds have been successfully developed to provide guidance and biological cues to facilitate Schwann cell myelination and orientated axonal growth. Numerous assessment techniques have been employed to investigate the therapeutic efficacy of nerve scaffolds in promoting the growth of Schwann cells and axons upon the bioactivities of distinct scaffolds, which have encouraged a greater understanding of the biological mechanisms involved in peripheral nerve development and regeneration. However, it is still difficult to compare the results from different labs due to the diversity of protocols and the availability of innovative technologies when evaluating the effectiveness of novel artificial scaffolds. Meanwhile, due to the complicated process of peripheral nerve regeneration, several evaluation methods are usually combined in studies on peripheral nerve repair. Herein, we have provided an overview of the evaluation methods used to study the outcomes of scaffold-based therapies for PNI in experimental animal models and especially focus on Schwann cell functions and axonal growth within the regenerated nerve.

## 1 Background

Peripheral nerve injury (PNI) is a traumatic nervous disease in clinical settings with significant severity worldwide, which is commonly caused by accidents, war, and surgical complications (Houshyar et al., 2019; Meena et al., 2021; Chen et al., 2022; Hercher et al., 2022; Hui et al., 2022). It has been reported that peripheral nerve injuries affect approximately 2.8% of trauma patients annually, causing a serious health problem (Taylor et al., 2008; Vijayavenkataraman, 2020; Acheta et al., 2021). PNI can lead to lifelong disability in patients with neuropathic pain, depending on the severity of the injury (Yi et al., 2019; Davoli-Ferreira et al., 2020; Idrisova et al., 2022; Sharifi et al., 2023; Xiong et al., 2023). As the regeneration rate of damaged peripheral nerves is approximately 1 mm per day, the strategies and outcomes of clinical treatment on PNI usually depend on the different types of injury and the distances of the gaps (Seddon et al., 1943; Sunderland, 1947; Juckett et al., 2022; Zhang et al., 2023a; Choe et al., 2023). Moreover, patients may need multiple surgeries and extended hospitalizations, which places a financial burden on the healthcare system (Padovano et al., 2022; Dong et al., 2023).

Nowadays, the standard clinical intervention for bridging millimeter-scaled nerve gaps often relies on a tension-free end-to-end suture (Lopez-Cebral et al., 2017; Kou et al., 2019; Leite et al., 2019). However, for longer-gapped PNI, the distal nerve and end tissue may undergo atrophy over time, which can cause permanent sensory and motor dysfunction (Tang et al., 2021). The gold standard for treating long-gapped PNI is to bridge the nerve defect using an autologous nerve graft in the clinic (Heath and Rutkowski, 1998). Nevertheless, secondary injury, mismatch on the size of donor and recipient nerve tissues, and peripheral neurofibroma formation usually limit the therapeutic efficiency and outcome of autologous nerve grafts, which can result in poor sensory and functional recovery (Bombeiro et al., 2016; He et al., 2020; Manto et al., 2021; Tang et al., 2022). Thus, artificial nerve scaffolds have been explored to facilitate the desirable regeneration of nerves after injuries. In recent decades, artificial scaffolds, with effective guidance and biological cues to facilitate Schwann cell (SC) myelination and axonal growth, have emerged along with a deeper understanding of biological mechanisms in peripheral nerve development and regeneration (Jiang et al., 2022a; Zhang et al., 2022a; Huang et al., 2022; Smith et al., 2022; Thibodeau et al., 2022; Zhang et al., 2023b). However, when evaluating the effectiveness of novel artificial scaffolds, it is difficult to compare the results from different labs due to the diversity of protocols and the availability of innovative technologies. Therefore, in this review, we have explored the useful methods applied in evaluating the outcomes of scaffold-based therapies for PNI in experimental animal models and especially focus on Schwann cell functions and axonal growth within the regenerated nerve.

## 2 Schwann cells and peripheral nerve regeneration

### 2.1 Schwann cells

Schwann cells are the most well-known and abundant cell type of peripheral glial cells, which have been well-studied in peripheral nerve repair (Jessen and Arthur-Farraj, 2019; Wang et al., 2019; Wang et al., 2023a). With deeper understanding about the biological functions of Schwann cells in nerve repair, distinct populations of SCs have been revealed. Generally, SCs are divided into myelinating and non-myelinating cell types. Myelinating SCs ensheath axons to facilitate the conduction of electric impulses during nervous activity, while non-myelinating SCs, such as Remak SCs, surround axons of small caliber and provide trophic support to unmyelinated axons (Brifault et al., 2020; Bosch-Queralt et al., 2023; Procacci et al., 2023). In addition to forming myelin sheaths around axons and secreting neurotrophic factors, SCs can also regulate sensory perception and facilitate cell communications between synapses (Faroni et al., 2014). Moreover, it has been found that SCs also play a significant role in neuroinflammation and neuropathic pain (Wang et al., 2022; Adam et al., 2023; Chen et al., 2023).

### 2.2 Schwann cells in PNI repair

**SCHEME 1:**
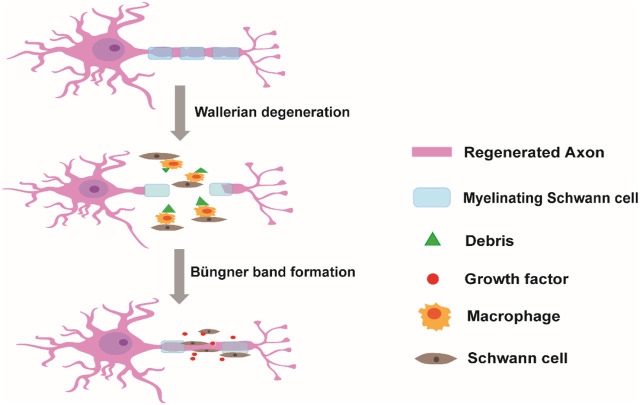
Schwann cells in the repair of peripheral nerve injury. After injury, the debris of injured axons in the distal stump is segmented and cleaned by Schwann cells and by recruited macrophages. Once the myelin debris is cleared, Schwann cells release a series of neurotrophic factors and guide axonal regrowth to the distal stump by forming Büngner bands.

All types of SCs can promote peripheral nerve regeneration through their cell differentiation and functional alteration after PNI. Soon after injury, demyelination of injured peripheral nerves occurs and axons break into debris in the distal stump via Wallerian degeneration (Zou et al., 2022). Then, SCs are activated in the repair of PNI. Initially, SCs segment the debris of injured peripheral nerves and induce macrophage recruitment to clean the debris to enable successful outgrowth of axons in the proximal stump. During the process of axonal regrowth, activated SCs differentiate into mature SCs and highly express myelination-associated genes, such as peripheral myelin protein 22 (PMP22), myelin basic protein (MBP), and myelin-associated glycoprotein (MAG). Meanwhile, mature SCs secrete various neurotrophic factors, such as nerve growth factor (NGF) and brain-derived neurotrophic factor (BDNF), to provide a supportive microenvironment for encouraging nerve growth. Moreover, SCs can also guide axonal regrowth to the distal stump by forming Büngner bands (Scheme 1) (Cristob and Lee, 2022; Talsma et al., 2022; Yuan et al., 2022).

### 2.3 Biomaterials on modulation of Schwann cell and axon behavior

**SCHEME 2:**
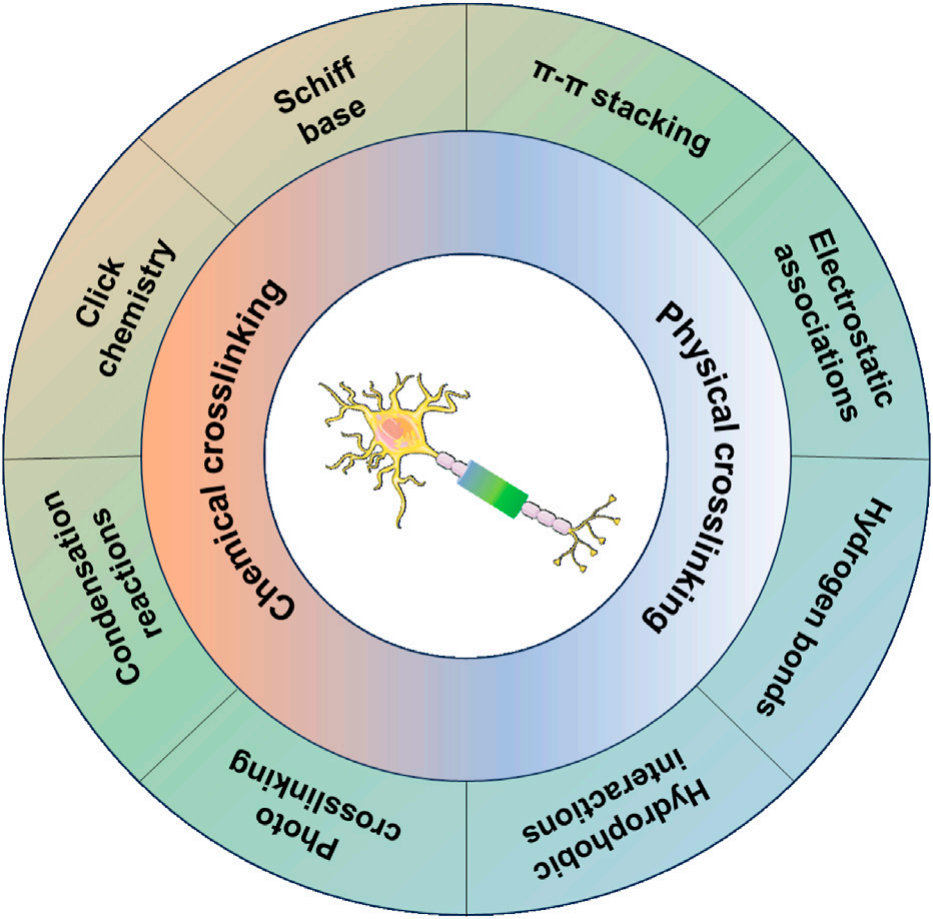
Chemical and physical crosslinking methods of fabrication of hydrogel conduits for promoting nerve growth.

Several studies and molecular mechanisms have demonstrated that effective modulation of Schwann cell and axon behavior is a promising strategy for PNI repair (Liu et al., 2019; Xuan et al., 2020; Huang et al., 2021). Recently, hydrogel conduits have been regarded as powerful tools for promoting PNI repair by mimicking the extracellular matrix (ECM) of nerve tissue and serving as carriers for delivering drugs, growth factors, and cells of various types (Liu et al., 2021; Rao et al., 2022; Arif et al., 2023). The ways to crosslink the polymer chains within hydrogel conduits determine the properties of conduits, such as stability, biodegradability, and release of bioactive molecules for modulating nerve regeneration. Normally, chemical crosslinking and physical crosslinking are the two main ways of fabricating hydrogel conduits. Chemical crosslinking methods include Schiff base, click chemistry, condensation reaction, or photo-crosslinking, which can equip the hydrogel conduits with improved mechanical strength and stable immobilization of bioactive molecules to match the mechanical properties of nerve tissue and achieve long-term bioactivity. On the other hand, physical crosslinking methods, such as electrostatic associations, π–π stacking, hydrogen bonds, and hydrophobic interactions, avoid the use and toxicity of crosslinkers in chemical crosslinked hydrogel conduits and draw much attention in the field of delivering growth factors for promoting nerve growth (Solomevich et al., 2023) (Scheme 2).

**SCHEME 3:**
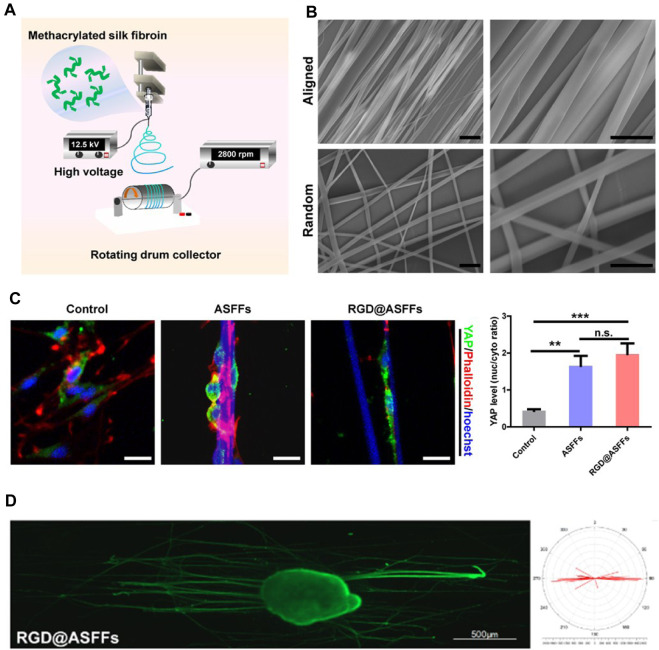
Aligned electrospun fiber-based scaffolds containing anisotropic topological cues that promote peripheral nerve regeneration by mimicking the microstructure of nerve tissue. **(A)** Schematic illustration of preparation of well-aligned electrospun fibers. **(B)** SEM images of aligned fibers. Scale bar = 10 μm. **(C)** The topology of aligned fibers facilitated the differentiation of Schwann cells by activating YAP nuclear translocation. **(D)** Aligned fiber scaffolds benefit orientated axon growth. (Figures reproduced from Chen et al., 2022).

To better enhance the therapeutic efficacy of nerve scaffolds, natural polymer-based hydrogels with ECM-associated proteins or peptides have been engineered to provide similar mechanical properties and bioactivities as the native tissues to maintain the biological functions of the SCs and accelerate axon growth (Joshi et al., 2023; Valentino et al., 2023). Aligned patterns have been created on the nerve scaffolds to guide the orientated growth and myelination of SCs (Wang et al., 2020; Gu et al., 2021). To encourage the growth of Schwann cells based on the aligned patterns, inducing newborn axonal sprouts from the proximal stump to the distal stump, poly(D,L-lactide-co-caprolactone) (PLCL) films with linear micropatterns of graphene oxide- or bioactive peptide-modified microscaled ridges and grooves have been developed to promote the aligned migration of Schwann cells to facilitate peripheral nerve regeneration (Zhang et al., 2020; Zhang et al., 2022b). Furthermore, the aligned electrospun fiber-based scaffolds containing anisotropic topological cues at the micro–nanoscale have also been fabricated to preciously mimic the microstructure of peripheral nerve tissue to improve the guidance and biological cues for supporting nerve regeneration (Yi et al., 2020; Cavanaugh et al., 2022; Chen et al., 2022) (Scheme 3).

## 3 *In vivo* evaluation of nerve regeneration and functional recovery

**SCHEME 4:**
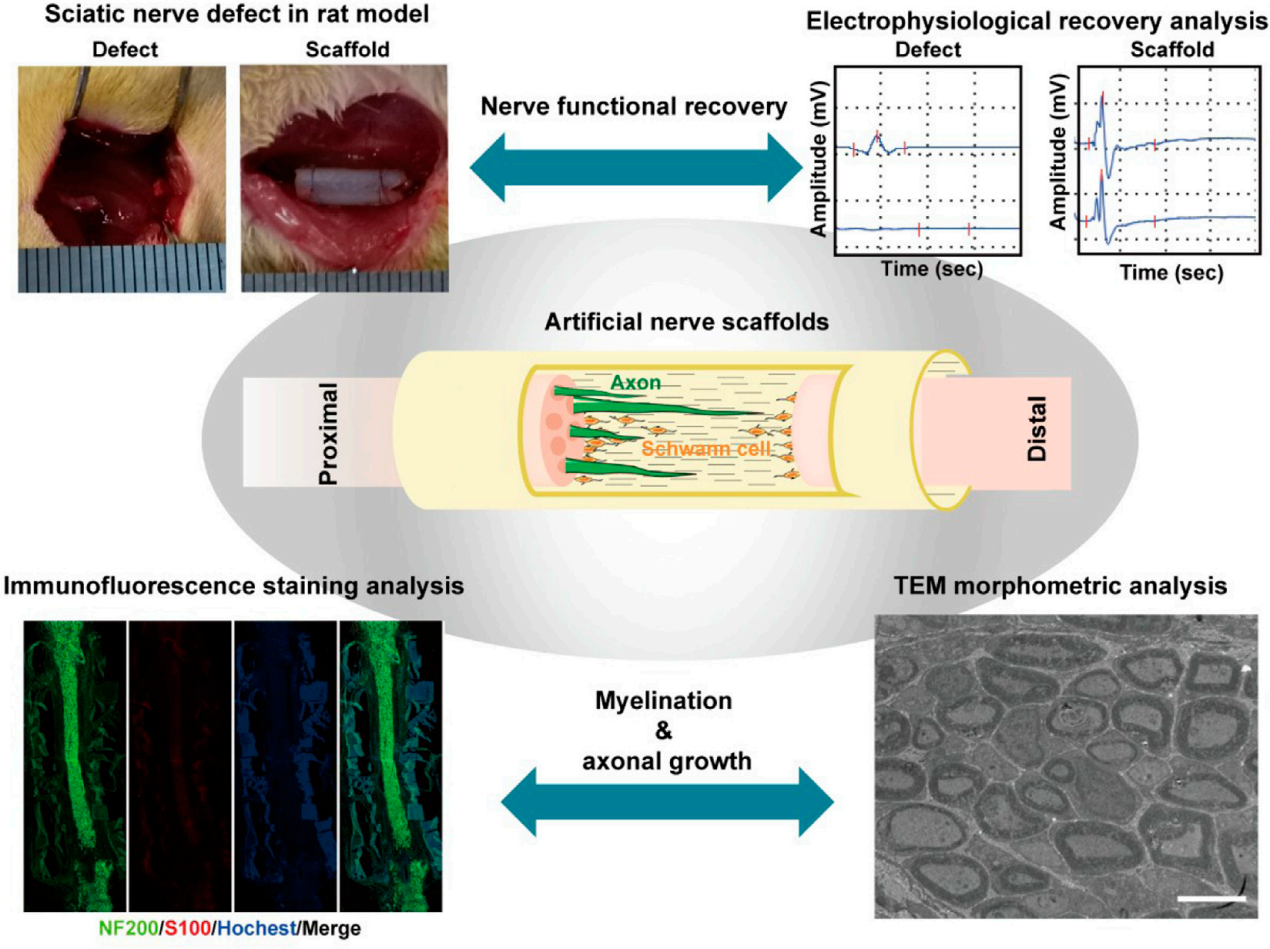
*In vivo* evaluation methods on nerve regeneration and functional recovery in sciatic nerve defect rats treated by artificial bioscaffolds, including electrophysiological recovery analysis of the muscular response to electrical nerve stimulation, histological examination of regenerated nerve tissue, and morphometric analysis of the regenerated nerve by TEM. (Figures reproduced from Chen et al., 2022; Tang et al., 2021).

Various evaluation methods have been used to study the therapeutic effect of nerve scaffolds on facilitating the growth of SCs and axons *in vitro* and *in vivo*, depending on the biological functions of different scaffolds. Among them, evaluations of the locomotor behavior of animals, electroneurography of nerves, weight loss of muscles, and histological examination of regenerated nerve tissue have been considered gold standards for analyzing the *in vivo* therapeutic effect of bioscaffolds on promoting nerve regeneration and functional recovery in current studies (Table 1) (Liu et al., 2022; Duan et al., 2023; Fang et al., 2023; Gao et al., 2023; Jin et al., 2023; Li et al., 2023; Semmler et al., 2023; Wu et al., 2023; Yan et al., 2023; Yang et al., 2023). These studies demonstrated proper methods to assess the degree of nerve regeneration and events occurring during the nerve repair process. These experimental studies allow us to determine the effects of biomaterial-based therapies and compare different strategies for promoting PNI repair. Benefiting from well-designed evaluation methods, specific questions regarding bioscaffold-promoted nerve regeneration can gradually be answered via a combination of various methods, and a better way can be found to design improved and multi-functional bioscaffolds from the appropriate information (Scheme 4).

**TABLE 1:** *In vivo* analysis and outcomes using artificial nerve scaffolds compared to autograft.

Conduit	Species	Nerve gap (mm)	Time point	*In vivo* analysis	Outcome compared to autograft (%)	Diameter of myelinated nerve fiber	Reference
Silk fibroin and gelatin methacryloyl	Sprague–Dawley rats	12	16 weeks	Electrophysiological recovery/myelinated nerve fibers/myelin thickness	94	5.21 ± 1.35 μm	Wu et al. (2023)
Silk fibroin and spider dragline silk	Sprague–Dawley rats	10	14 weeks	Footprint analysis/myelinated nerve fibers/axonal diameter	95	4.76 ± 1.52 μm	Semmler et al. (2023)
Gelatin methacryloylate	Sprague–Dawley rats	5	12 weeks	Footprint analysis/electrophysiological recovery/myelinated nerve fibers/axonal diameter	69	8.10 ± 0.31 μm	Gao et al. (2023)
Hyaluronic acid and brain-derived neurotrophic factor and laminin	Sprague–Dawley rats	10	16 weeks	Electrophysiological recovery/myelinated nerve fibers/axonal diameter/myelin thickness	81	6.09 ± 0.65 μm	Yang et al. (2023)
Sodium alginate and decellularized porcine sciatic nerve	Sprague–Dawley rats	10	8 weeks	Footprint analysis/electrophysiological recovery/axonal diameter	90	—	Jin et al. (2023)
Chitin and rat adipose-derived mesenchymal stem cells	Sprague–Dawley rats	10	12 weeks	Footprint analysis/electrophysiological recovery/myelinated nerve fibers/axonal diameter/myelin thickness	93	5.73 ± 0.50 μm	Li et al. (2023)
Graphene oxide quantum dots and polycaprolactone	Sprague–Dawley rats	10	16 weeks	Footprint analysis/electrophysiological recovery/axonal diameter/myelin thickness	81	9.15 ± 1.60 μm	Yan et al. (2023)
Polycaprolactone and graphene oxide and type I collagen nanofibers	Sprague–Dawley rats	10	8 weeks	Footprint analysis/myelinated nerve fibers/myelin thickness	97	4.8 ± 1.08 μm	Fang et al. (2023)
Type I collagen and mineralized collagen	Sprague–Dawley rats	10	12 weeks	Footprint analysis/electrophysiological recovery/myelinated nerve fibers/myelin thickness	82	4.62 ± 0.34 μm	Duan et al. (2023)
Chitosan and Wnt5a and thrombin	Sprague–Dawley rats	10	12 weeks	Footprint analysis/electrophysiological recovery/myelinated nerve fibers/axonal diameter/myelin thickness	87	3.45 ± 0.26 μm	Liu et al. (2022)
Poly (D,L-lactide-co-caprolactone) and CQAASIKVAV peptide	Sprague–Dawley rats	10	16 weeks	Electrophysiological recovery/myelinated nerve fibers/axonal diameter/myelin thickness/	89	5.43 ± 0.69 μm	Zhang et al. (2022b)

### 3.1 Walking track analysis

Walking track analysis is usually conducted to evaluate the nerve functional recovery of a rat model after conduit implantation. During the experiment, rats are made to walk, and their footprints are recorded at the CatWalk system. For example, Wang et al., 2023 developed CYIGSR functionalized conduits to bridge 10-mm rat sciatic nerve defects, and the nerve function recovery of rats was evaluated by footprint analysis at 2 weeks post-treatment. The sciatic nerve function index (SFI) of rats in the CYIGSR functionalized conduit group was higher than that in the chitosan control group, indicating that immobilization of CYIGSR on nerve scaffolds can significantly promote nerve function recovery in PNI (Wang et al., 2023b).

### 3.2 Video gait and ankle angle analysis

Measurement of active ankle angle at the terminal stance (ATS) is another way to assess the nerve functional recovery after bioscaffold-based treatment. Lee et al. measured the ATS of rats every 3 weeks during 12 weeks post-surgery and photographed them with a 60 Hz digital camera positioned at a distance of 1 m to the animal. Rats treated with poly(lactide-co-ε-caprolactone) conduits containing NGF-loaded gelatin hydrogel showed gradual recovery of nerve function and exhibited improved ATS during 12 weeks, which was comparable to the autograft group, indicating the NGF sustainedly released from the conduits can accelerate nerve regeneration *in vivo* (Lee et al., 2022).

Although a large number of surgical interventions performed on patients are for the upper limb level in the clinic, sciatic nerve defect in the rat model is still dominant in experimental research. Therefore, most of the methods have been developed to assess the nerve function restoration of hindlimbs for evaluating the therapeutic effect of bioscaffolds. Walking track and ankle angle analyses have long been used in the assessment of sciatic functional nerve recovery. However, the validity of the sciatic functional index has been questioned, and it is important to find new ways to more accurately evaluate nerve functional recovery in rodents.

### 3.3 Electrophysiological recovery analysis

The restoration of electrical nerve stimulation from motor neurons to the muscles is essential for repair of nerve tissue. Thus, the muscular response to the electrical nerve stimulation is usually tested by electrophysiological recordings before the animals are sacrificed for histological analysis. Motor unit number estimation (MUNE) is a non-invasive electrophysiologic method to monitor the muscular response to electrical nerve stimulation by McComas et al. (1971). In the study by Cicero et al., electrophysiological recordings were conducted to assess the muscular response to electrical nerve stimulation at 120 days after implantation of nerve conduit in rats with a pair of mono-polar recording needle electrodes placed at the belly and the tibialis anterior (TA) and the gastrocnemious (GA) medialis muscles. The sciatic nerve was stimulated with square-wave pulses of 0.1-ms duration, and the compound muscle action potential (CMAP) response was recorded. Compared to the contralateral uninjured nerve, a significant decrease in MUNE in the operated limb was found. Meanwhile, the recovery of MUNE in the TA muscle of rats was much better in the poly-butylene succinate scaffold group compared to the untreated group, which was comparable to the healthy limb muscle, indicating that the poly-butylene succinate scaffolds effectively guided peroneal fibers (Cicero et al., 2022).

### 3.4 Recovery rate of gastrocnemius wet weight

In PNI, the denervated muscle undergoes atrophy and loss of muscle mass. When the muscle is innervated, the degeneration of the muscle can be interrupted and the muscle can recover its tropism (Ai et al., 2019; Rahmati et al., 2021). Therefore, the evaluation of the wet weight of the innervated gastrocnemius muscle reflects the index of nerve regeneration. Gastrocnemius muscles are usually harvested, and the wet weight of the muscle at the surgical site is compared with that at the non-surgical site. In Zaminy et al. (2021), 7-mm sciatic nerve defect in rats was bridged by using a decellularized nerve xenograft, and the recovery rate of the wet weight of the gastrocnemius muscle was significantly higher in the nerve autograft and acellular nerve xenograft groups without a significant difference at 8 weeks after surgery, demonstrating the promising capacity of acellular nerve xenografts on repairing sciatic nerve defects *in vivo*.

### 3.5 Histopathologic evaluation of regenerated nerve tissue

Immunofluorescence staining is a typical method to effectively visualize myelination, angiogenesis, axonal extension, and immune state within regenerated nerve tissue for evaluating bioscaffold-promoted nerve regeneration. S100β expression is a marker commonly used to assess glial cells in nerve repair, such as SCs in peripheral nerves; NF200 is a marker protein for assessment of nerve filaments. Dong et al. developed an aligned P(MMD-co-LA) fibrous membrane conduit loaded with deferoxamine (A_PDPLA/DFO) to bridge a 10-mm sciatic nerve defect in rats. Nerve paraffin sections were performed to detect axonal regeneration via immunofluorescence staining with a double staining of NF200 and S100β. At 24 weeks post-surgery, an identical nerve structure was visualized via the fluorescence of NF200 and S100β in the A_PDPLA/DFO group, and the positive areas of NF200 and S100β within the regenerated nerve were comparable to the autograft group, indicating that the release of deferoxamine from the A_PDPLA/DFO conduit successfully accelerated the process of nerve repair by promoting cell migration and guiding polarization of the vasculature (Dong et al., 2022).

The expression level of Ki67 in the regenerated nerve can reflect the proliferation state of axons, and the apoptotic state of axons can be evaluated using immunofluorescence staining of C-caspase3 in nerve tissues. Jiang et al., by immunofluorescent staining of Ki67 and C-caspase3, found that the protein level of Ki67 in the regenerated nerve tissue within the multilayered melatonin/reduced graphene oxide/polycaprolactone (MLT/RGO/PCL) conduit was significantly higher than that in the autograft and the protein level of C-caspase-3 was very low in the MLT/RGO/PCL group, indicating that the combination of MLT and RGO enhanced the axonal regrowth and reduced cell apoptosis in PNI repair (Jiang et al., 2022b).

The inflammatory response during the process of PNI repair greatly affects the outcome of bioscaffold-based treatment. To assess the infiltration of inflammatory cells and demyelination within the nerve tissues, Jahromi et al. collected the sciatic nerve tissues from 10-mm sciatic nerve defects in rats treated with nerve conduits containing Schwann cells and curcumin at 12 weeks post-surgery and stained them with hematoxylin and eosin (H&E) and Luxol fast blue (LFB). The histopathologic evaluation of nerve tissues indicated that incorporating Schwann cells and curcumin in nerve conduits significantly promoted sciatic nerve regeneration and neovascularization with minimal inflammatory responses (Jahromi et al., 2020)

However, there are some limitations in histopathologically evaluating regenerated nerve tissue via immunostaining and histochemical staining. As the aim of each experiment is different, certain dyes must be chosen carefully for single or even multiple tracing according to the interest of researchers in specific regeneration events. The results are largely based on the fixation methods and working conditions such as temperature and humidity. Moreover, extreme care must be taken for the quantitative analysis of nerve fibers and protein expression in the regenerated nerve tissue through immunofluorescence staining assays. Appropriate quantification in immunofluorescence-based methods usually depends on image acquisition and comparing the results to appropriate positive and negative controls to eliminate the errors.

### 3.6 Myelinated nerve fiber analysis by transmission electron microscopy

Morphometric analysis of ultrathin sections of the regenerated nerve is another effective way to determine the diameter of myelinated nerve fibers and the thickness of myelin sheaths. Zhu et al. cut tissue sections from the middle part of the regenerated nerves and stained them with uranyl acetate and lead citrate. By using transmission electron microscopy (TEM) evaluation, they found compact and uniformly structured myelinated nerve fibers in the ECM scaffold. The myelin sheathes of myelinated nerve fibers were relatively dense with intact basal membranes, indicating that ECM scaffolds with microchannels held a strong capacity for guiding nerve regeneration and prompting functional recovery (Zhu et al., 2019). In addition to analyzing the mean diameter of myelinated nerve fibers and myelin sheath thickness, Amini et al. (2020) also imaged cross-sections of the nerve conduit after implantation by TEM and calculated the G-ratio by comparing the inner axonal diameter to the total outer diameter for determining the speed of fiber conduction. Thicker axonal diameter of myelinated nerve fibers was found in regenerated nerves with a G-ratio of 0.6 in PCL with 15% of the lignin nanoparticle conduit group, indicating the dominant role of lignin in nerve repair (Amini et al., 2020).

### 3.7 Expression of neurotrophic factors

SCs can create a favorable microenvironment for axonal growth by releasing neurotrophic factors, which plays an important role in PNI recovery. Therefore, it is of great importance to evaluate the capacity of bioengineered nerve conduits in promoting neurotrophic factor release from SCs *in vivo*. In Yang et al.’s study, the sciatic nerve tissue in conduits was harvested and transferred immediately into liquid nitrogen for further RNA extraction, and the gene expression of neurotrophic factors, vascular endothelial growth factor (VEGF), and myelin genes was evaluated by using the qRT-PCR assay. It was found that rats in peptide hydrogel groups exhibited significantly enhanced gene expression of NGF, BDNF, VEGF, and PMP22, indicating that dual-functionalized peptide hydrogels of RAD/IKV/RGI promoted SC myelination and development to facilitate growth factor secretion for nerve regeneration (Yang et al., 2020). Mao et al. assessed the changes in key factors (NGF and VEGF) that contribute to nerve regeneration in regenerated nerve tissues using Western blotting. Results showed that the conduit with piezoelectric stimulation effectively promoted NGF and VEGF expression in nerve tissues at 28 days post-surgery, indicating that piezoelectric stimulation of the conduit had a promising therapeutic effect on PNI repair by enhancing neurotrophic factor secretion (Mao et al., 2022).

#### 3.7.1 Future perspectives

Overall, the treatment for large-gapped PNI is still suboptimal, which affects patients’ quality of life and causes significant healthcare costs. The artificial scaffolds can modulate Schwann cell behavior and encourage axonal regrowth in nerve regeneration by providing various physical and biological cues to create a favorable microenvironment, which have emerged as alternative tools for prompting PNI recovery. In this review, we have focused on discussing experimental methods for evaluating Schwann cell behavior and axonal extension regulated by bioscaffolds in PNI repair. Since the process of peripheral nerve regeneration is very complicated and involves various cell types, such as SCs, neurons, fibroblasts, macrophages, and even T cells, there is no single evaluation method that can comprehensively answer all the questions in bioscaffold-based therapy. Therefore, it is important to combine appropriate evaluation methods to study the therapeutic effect of novel biomaterials in terms of animal functional behavior, morphological and histopathologic evaluation of regenerated nerves, and analyses of growth factors at the biomolecular level.
